# How does HIV-related stigma correlate with HIV prevalence in African countries? Distinct perspectives from individuals living with and living without HIV

**DOI:** 10.1186/s12889-023-16545-3

**Published:** 2023-09-04

**Authors:** Arlette Simo Fotso, Connor G. Wright, Andrea Low

**Affiliations:** 1 L’Institut national d’études démographiques (INED), F-93300 Aubervilliers, France; 2 Centre Population & Développement, Université Paris-Cité, Inserm, Paris, France; 3 ICAP at Columbia University, Mailman School of Public Health, Columbia University, New York, NY USA

**Keywords:** Stigma, HIV Prevalence, PHIA, People living with HIV, Africa

## Abstract

**Background:**

Population-level research evaluating HIV-related stigma among countries with varied national HIV prevalence is scarce. To better understand HIV-related stigma and mitigate its potential negative effects, it is necessary to evaluate its relationship with HIV prevalence, as well as the mechanisms that influence it. This study aimed to analyze how HIV-related stigma correlates with subnational HIV prevalence in three African countries with varied HIV epidemics.

**Methods:**

This paper used data from the nationally representative Population-based HIV Impact Assessment (PHIA) surveys conducted from 2015–2017 in Malawi, Zambia, and Tanzania. Each country's sub-national geographic divisions were used to categorize them as low (0–5.4%), middle (5.5–11.2%), and high (11.3–17.1%) HIV prevalence regions in the main analysis. Questions from the survey stigma module were used to measure HIV-related stigma. Logistic regression and multilevel models were performed to assess the associations between the level of sub-national HIV prevalence and HIV-related stigma measures among persons living with, and without, HIV.

**Results:**

The results show that the odds of people living without HIV expressing stigmatizing behavior towards PLWH was significantly lower in regions of middle (OR = 0.80, 90%CI = (0.68–0.96)) and high (OR = 0.65, 90%CI = (0.53–0.80)) HIV prevalence when compared to low prevalence regions. The odds of reporting discriminatory attitudes were also lower for those in middle (OR = 0.87, 90%CI = (0.78–0.98)) and high (OR = 0.64, 90%CI = (0.56–0.73)) HIV prevalence regions compared to others. Living in middle and high HIV prevalence regions was associated with lower odds of expressing prejudice toward PLWH (OR = 0.84, 90%CI = (0.71–0.99) and OR = 0.60, 90%CI = (0.45–0.80), respectively) among people living without HIV. Notably, PLWH living in high prevalence regions had higher odds of reporting internalized stigma (OR = 1.48, 90%CI = (1.02–2.14)) compared to those living in low prevalence regions.

**Conclusions:**

The results indicate that among people not living with HIV, subnational HIV prevalence was negatively associated with discriminatory attitudes and prejudice towards PLWH, but HIV prevalence was positively associated with self-reported internalized stigma among PLWH. These results provide insight on how resources could be invested to reduce HIV related stigma among both PLWH and those not living with HIV.

**Supplementary Information:**

The online version contains supplementary material available at 10.1186/s12889-023-16545-3.

## Background

Research on HIV/AIDS-related stigma and discrimination has found significant associations between high stigma environments and negative health outcomes. High levels of self-reported stigma have been reported to strongly correlate with depressive and psychosocial symptoms [1–4], treatment non-adherence [1, 3, 4], low community-level and individual-level HIV testing behaviors [5, 6], non-disclosure of HIV status [7], low social support [8, 9], employment barriers [10] and low income levels [8]. The growing science surrounding negative health outcomes and HIV/AIDS-related stigma has justified the need to effectively understand the complexities of these shifting mechanisms and integrate them into HIV prevention, treatment, and care programs to innovate efforts to reduce the progression of the HIV pandemic as defined in the 2025 UNAIDS AIDS Targets [11].

There has been substantial research on HIV/AIDS-related stigma among PLWH [12–15] and key-populations with concentrated epidemics [16–19], however there has been less population-level research evaluating HIV/AIDS-related stigma across national contexts with varied HIV prevalence [20–23]. To understand these diverse HIV/AIDS epidemics it is necessary to evaluate the relationship between HIV prevalence and self-reported HIV-stigma among people living with, and without, HIV as well as the mechanisms that influence it. People living without HIV, often in positions of power in a society, can perpetuate stigma in various forms. Stigma can manifest via prejudice, stereotypes, discriminatory attitudes, and stigmatizing behaviors, which operate together to maintain societal level stigma [14, 24]. These manifestations could be derived from fear of seroconversion or ideas of death [22, 24] or rooted behind a lack of HIV/AIDS knowledge [21] among people living without HIV. Conversely, PLWH can experience stigma through different manifestations: enacted, or experienced stigma, anticipated stigma, and internalized and perceived stigma [14, 24]. 

Manifestations of HIV/AIDS-related stigma and discriminatory attitudes have been found to differ between countries with high and low/middle HIV prevalence [20, 21]. Genberg et al. compared negative attitudes and perceived acts of discrimination towards PLWH in four countries. They reported the highest negative attitudes towards PLWH were found in the lowest HIV prevalence countries [21], however the objectives of this study did not include the evaluation of experienced or internalized HIV stigma mechanisms. Additionally, this study only evaluated a small sample of communities in Tanzania, Zimbabwe, South Africa, and northern Thailand, thus cannot provide insight on nationally representative HIV-related stigma data compared to HIV prevalence. Du et al. furthered this understanding by comparing country-level HIV prevalence with country-level and individual level stigma in 42 countries, suggesting that national HIV prevalence has an impact on individual-level attitudes toward PLWH [20]. These two studies highlight the importance of considering the magnitude of each countries HIV epidemic distinctly to design efficient policies and interventions.

Du et al. was one of the first studies to further evaluate the mechanisms involved between a higher national HIV prevalence and its negative association with HIV stigma. The researchers hypothesized that countries with high HIV prevalence are more likely to use resources in promotion of HIV education, as well as laws and policies created to address HIV misconceptions and stigma. Thus, countries with higher HIV prevalence would be associated with lower levels of HIV-related stigma. The study also found that HIV prevalence was positively associated with HIV knowledge and AIDS-related spending. However, as potential mediators, they found that HIV knowledge, but not AIDS spending, negatively predicted HIV-related stigma [20]. The study is limited by a single-item measure of stigma, which hindered their ability to capture a comprehensive understanding of all types of stigma, including the above three mechanisms PLWH experience stigma [14, 24]. Furthermore, the authors only used country-level HIV prevalence, which could hide the heterogeneity of HIV burden within countries. In fact, in Sub-Saharan Africa, HIV prevalence varies significantly within national borders. Between 2015 and 2017, adult HIV national prevalence was 4.9% (subnational prevalence ranging from 0 to 11%) in Tanzania, 12% (subnational prevalence ranging from 6 to 16%) in Zambia and 11% (subnational prevalence ranging from 5 to 18%) in Malawi [25–27]. There is therefore a necessity for further research that can evaluate population-level HIV data and more HIV-related stigma measures at the subnational and individual level.

The rational for evaluating stigma measures at the sub-national are to investigate localized behaviors and social environments. Sub-national levels can provide a greater granularity to the socioeconomic and demographic differences within a country, which may interact with their social environment. For example, if stigma is perpetuated by fear of seroconversion or ideas of death, as mentioned previously [22, 24], interaction with communities living with HIV impact this fear, thus making it more relevant to evaluate sub-national HIV prevalence rather than national prevalence. Conversely, if sub-national HIV prevalence is higher, PLWH may have a more robust social network that could impact internalized, anticipated, and expressed stigma at this level.

To further understand the association between HIV prevalence and stigma, this study used individual-level stigma measures from three Population-based HIV Impact Assessment (PHIA) surveys, to analyze how HIV-related stigma and discrimination correlate with subnational HIV prevalence in three African countries. In doing so, this article distinguished between the perspectives of self-reported PLWH and people self-reporting not living with HIV. PHIA surveys’ primary objective was to provide population-level assessment of the burden of HIV disease at the national and sub-national level (as defined by census data in each country), and to document the achievement of HIV programs in participating countries [28]. Detailed results on PHIA primary and secondary objectives, including on testing and knowledge of HIV status, can be found in countries’ PHIA survey respective reports [25–27].

## Methods

### Data

This study used data from the PHIA surveys conducted in Malawi, Zambia, and Tanzania between 2015 and 2017 [25–27]. The household-based national surveys were conducted by the Ministries of Health, with funding from the U.S. President’s Emergency Plan for AIDS Relief (PEPFAR), technical assistance from the U.S. Centers for Disease Control and Prevention (CDC) and implemented by ICAP at Columbia University.

The PHIA surveys are cross-sectional household-based surveys using a stratified multistage (enumeration region and household) survey sampling design, with strata defined by sub-national geographic divisions used in each country’s latest census [28]. More information on sub-national calculation and associated sub-national data can be found on the PHIA website [28]. The primary objectives of the PHIA surveys were to estimate national HIV incidence and sub-national prevalence of viral load suppression, defined as HIV RNA < 1000 copies/ml, among adults. Participants residing in the household for the past two nights were assigned as residents of that household, and their data corresponds with the respective sub-national region. Participants were asked to provide their HIV status, and blood specimens were collected for home-based HIV testing and counseling (HBTC) and point-of-care CD4 + T-cell enumeration with immediate return of results. Blood samples from PLWH underwent laboratory-based confirmatory testing, HIV incidence testing, RNA polymerase chain reaction (viral load), DNA polymerase chain reaction (early infant diagnosis), and serum antiretroviral drug detection [29]. The results of each PHIA survey are available on the PHIA website, including national and sub-national HIV prevalence, HIV incidence, and VLS [28]. Additionally, each survey collects socio-ecological information, including a module with questions referring to individual and community-level HIV/AIDS stigma among adolescents and adults (dependent on each survey). The current study focusses on the adult population aged 15 to 49 years.

### Variables

#### Independent variables

Our variable of interest, the weighted HIV prevalence among adults aged 15–49 years, was measured at the subnational level, referred to as a region, zone or province depending on the country. This allowed us to account for the heterogeneity of the burden of disease inside these countries. We transformed the variable of interest into a categorical variable indicating whether individuals belong to subnational administrative entities with a low (prevalence between 0–5.4), middle (prevalence between 5.4–11.2) and high (prevalence 11.2 -17.1) HIV prevalence. The cutoff was based on tercile of the distribution (unweighted) of the subnational HIV prevalence.

A set of socio-demographic individual-level characteristics were used as the control variables in this analysis, including age, sex, education, and wealth quintile. Subnational-level controls included region, education (measured as the proportion of individual with more than secondary education), and wealth (defined as the proportion of individuals within the highest wealth quintile).

#### Outcome variables: HIV related stigma measurements

The manifestations of HIV-related stigma considered in this analysis follow the health stigma and discrimination framework published by Earnshaw et al. and further developed by Stangl et al., [14, 24]. Questions from the stigma module were leveraged to measure discriminatory attitudes, stigmatizing behavior, and prejudice among participants not living with HIV towards PLWH. Questions from the same module, when asked to PLWH, captured experienced stigma and discrimination, internalized stigma, and anticipated stigma. The two following questions on discrimination were asked: “Would you buy fresh vegetables from a shopkeeper or vendor if you knew that this person had HIV?”, and “Do you think that children living with HIV should be able to attend school with children who are HIV negative?” (Table 1). If a person who self-reported not living with HIV responded “no” to both questions, he/she was considered to display stigmatizing behavior and discriminatory attitudes, respectively, toward PLWH in this survey. In our conceptual framework, stigmatizing behavior refers to exclusion, avoidance or gossip, while discriminatory attitudes are beliefs that people with a specific condition should not be allowed to fully participate to the society [14, 20, 24]. Persons not living with HIV were considered to display prejudice if they agreed with the following statement, “I would be ashamed if someone in my family had HIV”. This question captures prejudice as defined as a negative evaluation the group and its members have towards another group, such as shaming of PLWH [24, 28].

**Table 1:** Measurements of HIV-related stigma

HIV related stigma	Question used	Response
	**Self-reported People living without HIV**	
Stigmatizing behavior	“Would you buy fresh vegetables from a shopkeeper or vendor if you knew that this person had HIV?”	No
Discriminatory attitude	“Do you think that children living with HIV should be able to attend school with children who are HIV negative?”	No
Prejudice	“I would be ashamed if someone in my family had HIV	Agree
	**Self-reported People living with HIV**	
Internalized stigma	“Would you buy fresh vegetables from a shopkeeper or vendor if you knew that this person had HIV?”	No
	“Do you think that children living with HIV should be able to attend school with children who are HIV negative?”	No
	“I would be ashamed if someone in my family had HIV”	Agree
Experienced discrimination	“In the last 12 months, have you been denied health services including dental care, because of your HIV status?”	Yes
Anticipated stigma	“In the last 12 months, when you sought health care in a facility where your HIV status is not known, did you feel you needed to hide your HIV status?”	Yes

To acknowledge the different stigma experiences among PLWH and people not living with HIV, we examined stigma and discrimination manifestations expressed by self-reported PLWH separately [14, 24]. For these measures, if a respondent living with HIV responded “no” to any of the questions on discriminatory and stigmatizing behavior and agreed with the statement, “I would be ashamed if someone in my family had HIV”, they were considered to have expressed internalized stigma. Internalized stigma or ‘self-stigma’ is defined in our conceptual framework as the adoption, by the stigmatized, of negative societal beliefs and feelings, as well as the social devaluation, associated with their stigmatized status [14, 24].

PLWH were also asked whether they have been denied health services, including dental care, because of their HIV status. In this case if the participant responded “yes”, we considered it as experienced discrimination. In our conceptual model, stigma and discrimination refer to the experience of stigmatizing behaviors that fall within or outside the purview of the law [14, 24]. PLWH were also asked if they felt they needed to hide their HIV status when they sought health care in a facility where their HIV status was not known. In the case of affirmative responses, PLWH were considered to display anticipated stigma, reflecting our stigma conceptualization that defines anticipated stigma as expectations of bias being perpetrated by others if their health condition becomes known [14, 24].

### Data analysis

To answer our research question, we used a logistic regression with country fixed-effects to account for characteristic similarities in individuals from the same country, and to account for country-specific policies that can influence HIV-related stigma. Then, for our sensitivity analysis, we further accounted for the nested structure of observation through a multilevel model, with level 1 being individuals and level 2 being the first administrative subdivision of the country. Given the number of countries in this analysis is relatively small, to be considered as a single level of a multilevel model, this latter model also includes country dummies for country fixed-effects. Another set of sensitivity analyses used a continuous variable of subnational HIV prevalence.

All the figures were weighted to account for sampling probabilities, non-response and post-stratification to national population projections from the survey year based on age and sex, using the PHIA appropriated replicates and base weights, with Taylor series linearization methods to estimate robust variances [30]. For the multilevel model, for simplicity reasons, only the base weights were used. This latter was scaled as per Carle’s [31] recommendation for multilevel analysis so that the new weights sum to the level 2 sample size. The levels of significance of 1%, 5%, and 10% were reported for all results. The analysis was conducted with Stata 16.

## Results

### Descriptive statistics

Overall, a sample of 31,173 people not living with HIV and 3,838 PLWH were included in the study. Table 2 presents socio-demographic characteristics of the sample.

**Table 2:** Samples characteristics and country-level indices

	Country							
	Zambia		Malawi		Tanzania		Total	
	%	90%CI	%	90%CI	%	90%CI	%	90%CI
	**People living without HIV**							
Stigmatizing behavior	14.7	[13.7,15.8]	6.0	[5.4,6.6]	16.5	[15.5,17.5]	14.2	[13.5,14.9]
Discriminatory attitude	10.4	[9.6,11.2]	6.3	[5.8,6.9]	11.6	[10.9,12.4]	10.4	[9.9,10.9]
Prejudice	16.6	[15.7,17.7]	13.3	[12.5,14.1]	14.9	[14.3,15.5]	14.9	[14.4,15.3]
Age								
15–24 years	37.1	[35.8,38.4]	36.5	[35.1,37.9]	31.0	[30.2,31.7]	33.1	[32.5,33.7]
25–34 years	36.2	[35.0,37.4]	36.5	[35.2,37.7]	38.2	[37.4,39.0]	37.5	[36.9,38.1]
35–44 years	20.9	[20.0,21.9]	20.6	[19.6,21.6]	23.8	[23.0,24.5]	22.7	[22.1,23.2]
45 years +	5.8	[5.4,6.3]	6.5	[5.8,7.2]	7.1	[6.7,7.6]	6.8	[6.4,7.1]
Sex								
Female	55.2	[54.3,56.2]	56.7	[55.5,58.0]	56.0	[55.2,56.7]	56.0	[55.4,56.5]
Male	44.8	[43.8,45.7]	43.3	[42.0,44.5]	44.0	[43.3,44.8]	44.0	[43.5,44.6]
Education								
Less than primary	5.6	[4.8,6.6]	8.4	[7.6,9.2]	11.6	[10.9,12.4]	10.0	[9.4,10.5]
Primary	38.7	[37.1,40.4]	59.5	[57.9,61.2]	59.2	[58.2,60.3]	55.7	[54.9,56.5]
Secondary level or higher	55.6	[53.7,57.6]	32.1	[30.4,33.8]	29.1	[27.9,30.4]	34.4	[33.5,35.3]
Wealth quintile								
Lowest	15.7	[14.4,17.1]	15.9	[14.6,17.2]	16.7	[15.0,18.5]	16.4	[15.3,17.5]
Second	17.7	[16.5,19.0]	17.9	[16.6,19.2]	18.7	[17.3,20.2]	18.4	[17.4,19.4]
Middle	19.9	[18.4,21.5]	19.5	[18.3,20.8]	19.8	[18.6,21.0]	19.8	[18.9,20.6]
Fourth	21.4	[19.7,23.3]	21.3	[19.9,22.7]	21.4	[19.7,23.2]	21.4	[20.2,22.6]
Highest	25.3	[23.2,27.5]	25.5	[23.7,27.3]	23.4	[21.7,25.3]	24.1	[22.9,25.4]
Urban Area								
No	54.5	[51.6,57.4]	79.4	[76.9,81.6]	59.1	[55.9,62.3]	62.1	[59.9,64.3]
Yes	45.5	[42.6,48.4]	20.6	[18.4,23.1]	40.9	[37.7,44.1]	37.9	[35.7,40.1]
Subnational HIV prevalence								
Low	5.8	[5.0,6.8]	37.6	[35.6,39.5]	66.1	[64.0,68.1]	50.1	[48.6,51.6]
Mid	31.8	[29.8,33.9]	22.2	[20.4,24.2]	29.1	[27.2,31.2]	28.3	[27.0,29.7]
High	62.3	[60.2,64.4]	40.2	[38.3,42.1]	4.8	[4.0,5.7]	21.6	[20.7,22.5]
Total	6394		6005		18,774		31,173	
	**People living with HIV**							
Internalised stigma	15.7	[13.5,18.1]	11.5	[9.4,14.0]	14.0	[11.5,17.0]	13.8	[12.4,15.3]
Experienced discrimination	1.6	[1.1,2.3]	1.5	[1.0,2.1]	2.6	[1.6,4.1]	2.0	[1.5,2.5]
Anticipated stigma	10.4	[9.0,12.1]	5.5	[4.5,6.7]	12.4	[10.1,15.1]	9.7	[8.6,10.9]
Age								
15–24 years	9.5	[8.2,11.0]	8.1	[6.7,9.6]	10.6	[8.5,13.2]	9.5	[8.4,10.7]
25–34 years	31.7	[29.6,33.9]	32.4	[29.9,35.1]	29.2	[26.0,32.6]	30.9	[29.3,32.6]
35–44 years	43.3	[41.1,45.6]	43.1	[40.6,45.7]	43.2	[39.8,46.6]	43.2	[41.5,44.9]
45 years +	15.5	[13.9,17.2]	16.4	[14.5,18.4]	17.0	[14.5,19.9]	16.3	[15.1,17.7]
Sex								
Female	67.5	[65.3,69.6]	67.4	[64.9,69.8]	73.3	[70.3,76.1]	69.7	[68.2,71.2]
Male	32.5	[30.4,34.7]	32.6	[30.2,35.1]	26.7	[23.9,29.7]	30.3	[28.8,31.8]
Education								
Less than primary	5.2	[4.2,6.3]	12.6	[10.9,14.4]	15.7	[13.4,18.3]	11.5	[10.4,12.8]
Primary	38.5	[36.1,41.1]	64.6	[62.2,66.9]	70.4	[67.2,73.5]	58.9	[57.2,60.6]
Secondary level or higher	56.3	[53.5,59.1]	22.8	[20.8,25.0]	13.9	[11.5,16.7]	29.5	[27.9,31.2]
Wealth quintile								
Lowest	8.2	[6.8,9.9]	16.2	[14.0,18.8]	12.8	[10.5,15.6]	12.5	[11.2,13.9]
Second	11.7	[9.8,13.9]	17.0	[14.9,19.3]	19.0	[16.0,22.5]	16.2	[14.7,17.8]
Middle	19.7	[17.4,22.1]	18.2	[16.2,20.3]	26.8	[23.2,30.7]	22.0	[20.3,23.9]
Fourth	28.2	[25.2,31.5]	22.2	[19.8,24.9]	23.4	[20.3,26.8]	24.5	[22.8,26.3]
Highest	32.2	[28.5,36.1]	26.4	[23.5,29.4]	17.9	[14.8,21.6]	24.8	[22.9,26.9]
Urban Area								
No	37.7	[34.0,41.6]	73.8	[70.5,76.8]	52.2	[47.2,57.1]	54.3	[51.7,56.9]
Yes	62.3	[58.4,66.0]	26.2	[23.2,29.5]	47.8	[42.9,52.8]	45.7	[43.1,48.3]
Subnational HIV prevalence								
Low	2.5	[1.7,3.6]	17.6	[15.1,20.4]	45.4	[40.8,50.1]	23.9	[21.8,26.1]
Mid	20.7	[18.3,23.3]	17.2	[14.9,19.8]	41.0	[36.4,45.7]	27.6	[25.4,29.9]
High	76.8	[74.1,79.3]	65.2	[62.0,68.3]	13.6	[10.6,17.3]	48.5	[46.2,50.8]
Total*	1513		1453		883		3838	

^*^Total sample for internalized stigma in Zambia, Malawi and Tanzania are 753, 703 and 883 respectively. All percentages are weighted using PHIA individual or knowledge module weights

Among persons not living with HIV, 14% and 10% displayed stigmatizing behavior and discriminatory attitudes, respectively, toward people with HIV. The highest percentage of persons expressing both stigmatizing behavior and discriminatory attitudes was observed in Tanzania (16% and 12%, respectively) and the lowest in Malawi (6%). Overall, 15% of individuals showed some prejudice toward PLWH, with the highest percentage observed in Zambia. Among people self-reporting living with HIV, 14% of PLWH expressed internalized stigma. This ranged from 11% in Malawi to 16% in Zambia. Experienced and anticipated stigma were expressed by 2% and 10% of PLWH in this sample, respectively, with the highest prevalence in Tanzania.

In both seronegative and seropositive groups, women were more represented in our sample (56% and 70%, respectively), people who only attained a primary education were predominant (56% and 59%, respectively), as well as people from the highest wealth quintile (24% and 25% respectively). Age distribution was different between the two groups. Among persons not living with HIV, the 25–34 years age-group was the largest, while those 35–44 years made up the majority of PLWH. Contrary to what is observed among people not living with HIV, the majority (62%) of PLWH lived in urban regions.

### Bivariate and multivariable analysis

The bivariate analysis of the association between subnational HIV prevalence and stigma manifestations is shown in Table 3. Among those not living with HIV, we observed that subnational HIV prevalence was negatively significantly associated with stigmatizing behavior, discriminatory attitudes, and prejudice. Compared to participants living in geographic regions with low HIV prevalence, those living in high prevalence regions were less likely to express stigmatizing behavior (OR = 0.55, 90%CI = (0.49–0.62)), discriminatory attitudes (OR = 0.61, 90%CI = (0.53–0.69)) and prejudice (OR = 0.88, 90%CI = (0.80–0.96)). No significant association between HIV prevalence and internalized, experienced, and anticipated stigma expressed by PLWH was found overall, before controlling for demographics. However, in our bivariate analysis by country, there was a significant positive association between subnational prevalence and anticipated stigma in Malawi. Compared to PLWH living in low prevalence regions, those living in middle and high prevalence region had higher odds of reporting anticipated stigma (OR = 4.8, 90%CI = (1.36–16.91) and OR = 5.91, 90%CI = (1.72–20.26) respectively). Among persons not living with HIV, a significant negative association between HIV prevalence and stigma was observed for all the indicators and in all the countries, except for the prejudice measure in Malawi and Tanzania.

**Table 3:** Association between regional HIV prevalence and type of stigma: Overall and for each country

	(1)	(2)	(3)	(4)	(5)	(6)
	Stigmatizing behavior	Discriminatory attitude	Prejudice	Internalized stigma	Experienced discrimination	Anticipated stigma
	OR/(90% CI)	OR/(90% CI)	OR/(90% CI)	OR/(90% CI)	OR/(90% CI)	OR/(90% CI)
**Overall**						
Subnational HIV prevalence: (Low)						
Mid	0.88	0.94	1.02	0.79	1.74	1.19
	(0.78–1.00)	(0.84–1.05)	(0.94–1.11)	(0.53–1.17)	(0.71–4.25)	(0.79–1.77)
High	0.55***	0.61***	0.88**	1.19	0.90	0.85
	(0.49–0.62)	(0.53–0.69)	(0.80–0.96)	(0.87–1.64)	(0.39–2.08)	(0.60–1.19)
**Zambia**						
Subnational HIV prevalence: (Low)						
Mid	1.04	1.08	0.90	0.52	0.51	1.39
	(0.81–1.32)	(0.82–1.41)	(0.68–1.18)	(0.26–1.05)	(0.12–2.22)	(0.67–2.87)
High	0.64***	0.65***	0.62***	0.81	0.28	0.95
	(0.52–0.78)	(0.50–0.85)	(0.48–0.80)	(0.44–1.49)	(0.07–1.17)	(0.47–1.90)
**Malawi**						
Subnational HIV prevalence: (Low)						
Mid	0.93	0.91	1.06	0.95	3.34	4.80**
	(0.70–1.25)	(0.71–1.16)	(0.91–1.25)	(0.37–2.43)	(0.55–20.34)	(1.36–16.91)
High	0.88	0.68***	0.92	2.21	3.51	5.91**
	(0.69–1.12)	(0.53–0.86)	(0.77–1.10)	(0.93–5.28)	(0.63–19.41)	(1.72–20.26)
Observations	9825	9809	9801	8371	25,242	25,242
**Tanzania**						
Subnational HIV prevalence: (Low)						
Mid	0.83**	0.90	0.93	0.78	1.87	1.02
	(0.71–0.97)	(0.78–1.02)	(0.85–1.03)	(0.46–1.32)	(0.63–5.57)	(0.61–1.71)
High	0.45***	0.61***	0.79	1.11	1.00	0.83
	(0.35–0.58)	(0.49–0.78)	(0.62–1.01)	(0.65–1.89)	(0.23–4.44)	(0.46–1.48)

Significance levels: * *p* < 0.1, ** *p* < 0.05, *** *p* < 0.01. p are nominal *p*-values and have not been corrected for multiple-hypothesis testing. *OR* Odds ratios, *CI* Confidence interval. All the estimations are weighted using PHIA individual or knowledge module weights

Table 4 shows that after controlling for age, sex, education, socioeconomic status, urban region and country fixed-effects, compared to persons living without HIV in low prevalence regions, the odds of expressing stigmatizing behavior were lower for those living in middle (OR = 0.83, 90%CI = (0.73–0.94)) and high (OR = 0.73, 90%CI = (0.64–0.84)) prevalence regions. Similarly, among persons living without HIV, the odds of expressing discriminatory attitudes were lower for those living in middle (OR = 0.89, 90%CI = (0.80–0.99)) and high (OR = 0.67, 90%CI = (0.59–0.77)) prevalence regions compared to low prevalence regions. In addition, those living in high HIV prevalence regions were less likely to express prejudice toward PLWH than those in low prevalence ones (OR = 0.78, 90%CI = (0.70–0.88)).

**Table 4:** Logistic regression of HIV related stigma among people living without HIV

	(1)		(2)		(3)	
	Stigmatizing behavior		Discriminatory attitude		Prejudice	
	OR	(90% CI)	OR	(90% CI)	OR	(90% CI)
Age group: (15–24 years)						
25–34 years	0.67***	(0.61–0.73)	0.74***	(0.68–0.82)	0.76***	(0.70–0.83)
35–44 years	0.61***	(0.56–0.67)	0.62***	(0.55–0.70)	0.64***	(0.59–0.71)
45 years +	0.65***	(0.56–0.74)	0.58***	(0.49–0.69)	0.67***	(0.58–0.77)
Sex:(Female)						
Male	0.90**	(0.83–0.97)	1.05	(0.97–1.14)	1.13***	(1.06–1.20)
Education: (Less than primary)						
Primary	0.50***	(0.45–0.56)	0.58***	(0.52–0.64)	0.82***	(0.74–0.91)
Secondary level or higher	0.23***	(0.20–0.26)	0.32***	(0.28–0.37)	0.65***	(0.57–0.75)
Wealth quintile: (Lowest)						
Second	0.82***	(0.73–0.92)	0.75***	(0.66–0.85)	0.96	(0.86–1.07)
Middle	0.70***	(0.62–0.79)	0.66***	(0.57–0.75)	0.86*	(0.76–0.98)
Fourth	0.54***	(0.47–0.62)	0.60***	(0.51–0.71)	0.81**	(0.71–0.94)
Highest	0.42***	(0.35–0.50)	0.51***	(0.41–0.62)	0.80**	(0.69–0.94)
Urban area:(No)						
Yes	0.84**	(0.73–0.97)	0.73***	(0.64–0.84)	0.75***	(0.67–0.84)
Subnational HIV prevalence: (Low)						
Mid	0.83**	(0.73–0.94)	0.89*	(0.80–0.99)	0.94	(0.86–1.01)
High	0.73***	(0.64–0.84)	0.67***	(0.59–0.77)	0.78***	(0.70–0.88)
Proportion with secondary educ. or higher	1.00	(0.99–1.01)	1.01***	(1.01–1.02)	1.01***	(1.01–1.02)
Proportion in highest wealth quintile	1.00	(1.00–1.01)	1.00	(0.99–1.00)	0.99***	(0.99–1.00)
Country: (Zambia)						
Malawi	0.26***	(0.21–0.33)	0.58***	(0.46–0.73)	0.83*	(0.71–0.98)
Tanzania	0.78**	(0.64–0.94)	1.05	(0.87–1.26)	0.93	(0.80–1.07)

Reference category is in parentheses. Significance levels: * *p* < 0.1, ** *p* < 0.05, *** *p* < 0.01. p are nominal *p*-values and have not been corrected for multiple-hypothesis testing. *OR* Odds ratios, *CI* Confidence interval. All the estimations are weighted using PHIA individual or knowledge module weights. Reference category in parentheses

Table 5 shows that, everything being equal, the odds of expressing internalized stigma is statistically significant, at the 10% level, for PLWH located in high HIV prevalence regions (OR = 1.69, 90%CI = (1.07–2.66)) compared to their peers living in low prevalence ones. Though not significant, we also observed the odds of expressing experienced and anticipated stigma were higher for those living in middle and high HIV prevalence regions compared to those living in low prevalence regions.

**Table 5:** Logistic regression of HIV related stigma among people living with HIV

	(1)		(2)		(3)	
	Internalized stigma		Experienced discrimination		Anticipated stigma	
	OR	(90% CI)	OR	(90% CI)	OR	(90% CI)
Age group: (15–24 years)						
25–34 years	0.68	(0.44–1.04)	0.59	(0.20–1.77)	0.82	(0.53–1.26)
35–44 years	0.76	(0.52–1.13)	0.48	(0.18–1.32)	0.64*	(0.42–0.98)
45 years +	0.84	(0.53–1.33)	0.56	(0.18–1.70)	0.55**	(0.34–0.91)
Sex:(Female)						
Male	0.95	(0.73–1.25)	1.26	(0.79–2.00)	0.94	(0.68–1.31)
Education: (Less than primary)						
Primary	0.66*	(0.45–0.95)	0.79	(0.36–1.72)	0.97	(0.63–1.50)
Secondary level or higher	0.39***	(0.24–0.63)	0.71	(0.30–1.67)	0.94	(0.57–1.54)
Wealth quintile: (Lowest)						
Second	0.59**	(0.38–0.89)	0.73	(0.25–2.16)	0.79	(0.45–1.38)
Middle	0.63**	(0.43–0.92)	0.58	(0.20–1.66)	0.90	(0.55–1.47)
Fourth	0.68	(0.44–1.07)	0.38	(0.13–1.08)	1.06	(0.65–1.72)
Highest	0.96	(0.59–1.58)	0.84	(0.30–2.38)	1.88*	(1.10–3.23)
Urban area:(No)						
Yes	0.59**	(0.41–0.87)	1.33	(0.72–2.47)	0.73	(0.50–1.05)
Subnational HIV prevalence: (Low)						
Mid	0.85	(0.56–1.30)	1.82	(0.75–4.43)	1.45	(0.92–2.28)
High	1.69*	(1.07–2.66)	1.25	(0.54–2.91)	1.49	(0.87–2.53)
Proportion with secondary educ. or higher	0.99	(0.97–1.02)	1.01	(0.96–1.06)	0.97	(0.95–1.00)
Proportion in highest wealth quintile	1.01	(0.99–1.02)	0.99	(0.96–1.02)	1.02*	(1.00–1.04)
Country: (Zambia)						
Malawi	0.39**	(0.19–0.79)	1.08	(0.31–3.70)	0.25***	(0.12–0.52)
Tanzania	0.76	(0.44–1.31)	1.72	(0.52–5.72)	0.88	(0.48–1.61)

Reference category is in parentheses. Significance levels: * *p* < 0.1, ** *p* < 0.05, *** *p* < 0.01. p are nominal *p*-values and have not been corrected for multiple-hypothesis testing. *OR* Odds ratios, *CI* Confidence interval. All the estimations are weighted using PHIA individual or knowledge module weights. Reference category in parentheses

The analysis, when considering the nested structure of the data (Tables A1 and A2), shows very similar results. The odds of expressing stigmatizing behavior towards PLWH was significantly lower for persons not living with HIV living in middle (OR = 0.80, 90%CI = (0.68–0.96)) and high (OR = 0.65, 90%CI = (0.53–0.80)) prevalence regions compared to low prevalence ones. Compared to individuals living in low prevalence region, the odds of expressing discriminatory attitudes were lower for those living in middle (OR = 0.87, 90%CI = (0.78–0.98)) and high (OR = 0.64, 90%CI = (0.56–0.73)) prevalence regions. Similarly, living in middle and high prevalence regions was associated with lower odds of expressing prejudice toward PLWH (OR = 0.84, 90%CI = (0.71–0.99) and OR = 0.60, 90%CI = (0. 0.45–0.80) respectively). PLWH living in high prevalence regions were significantly more likely to expressed internalized stigma (OR = 1.48, 90%CI = (1.02–2.14).

Regarding the control variables, we observed that among participants not living with HIV, older groups (25 + years) expressed fewer stigmatic attitudes compared to those younger (15–24 years). Similarly, more educated and wealthier people not living with HIV expressed fewer stigmatic attitudes compared to those reporting less education and wealth. Living in urban areas was associated with lower odds of expressing stigmatic attitudes. There was no significant difference between men and women for all outcomes, except stigmatizing behavior. The higher the proportion of individuals with secondary, or greater, education in a region, the higher the odds people expressed discriminatory attitudes and prejudice toward PLWH. Conversely, the higher the proportion of people in the highest wealth quintile in a region, the lower the odds people expressed discriminatory attitudes and prejudice.

Among PLWH, a similar relationship was observed regarding age, education, urban location, and wealth quintile for internalized stigma, though the relationship is in some of the cases non-significant. For anticipated stigma, only some older age-groups (35–44 and 45 + years) expressed significantly less stigma, while people from the fifth wealth quintile expressed more stigma compared to those with less wealth.

A sensitivity analysis using a continuous variable of subnational HIV prevalence is presented in the annex (Tables A3 and A4) and shows similar results: subnational HIV prevalence was negatively associated with stigmatizing behavior (OR = 0.96, 90%CI = (0.95–0.98)), discriminatory attitudes (OR = 0.96, 90%CI = (0.95–0.98)), and prejudice (OR = 0.98, 90%CI = (0.96–0.99)) of persons not living with HIV toward PLWH. There was a positive association with internalized stigma (OR = 1.06, 90%CI = (1.01–1.11)) expressed by PLWH. A positive, but non-significant, association between subnational HIV prevalence and experienced and anticipated stigma experienced or expressed by PLWH was found.

## Discussion

The aim of this paper was to assess the relationship between HIV prevalence and stigma, distinguishing between the perspectives of PLWH and persons not living with HIV in three African countries to account for heterogeneity of HIV burden. We found that subnational HIV prevalence was negatively associated with stigmatizing behavior, discriminatory attitudes, and prejudice toward PLWH among those not living with HIV. On the other hand, HIV prevalence appeared to be positively associated with internalized stigma expressed by self-reported PLWH. No significant association was found between experienced and anticipated stigma expressed by PLWH and sub-national HIV prevalence.

The negative association between HIV prevalence and stigma observed among persons not living with HIV is similar to the relationship established in the general population in previous cross-national studies [20, 21]. Du et al. [20], who work on mediation, found that two main elements explained such results. First, the amount of funding invested in high prevalence contexts. In these areas, more resources are invested in the promotion of laws and policies against stigma. Second, knowledge about HIV: in high prevalence contexts, more people are exposed to education and knowledge related to HIV, which might explain the lower stigma toward PLWH.

The positive relationship we found between HIV prevalence and internalized stigma among PLWH highlights the limitations of assessing the association between the two elements in a general population, as results can be heavily driven by persons not living with HIV. The necessity to characterize the relationship between HIV prevalence and stigma in the population of PLWH was already underlined by Du et al. [20], though to the best of our knowledge this is the first to do so, especially at the sub-national level. It is also important to note that consequences of stigma are not the same for people living without, versus with, HIV [14, 24].

Literature has shown that fear of death and seroconversion is one reason that might lead individuals to express forms of stigma [22]. Our results among PLWH could be explained by the fact that this group has continued to associate HIV with death, suffering, and shame, and think persons not living with HIV feel similarly. In high prevalence regions, individuals could have more frequent contact with PLWH, hence PLWH might think that this fear is higher and thus express more internalized stigma. The results show that internalized stigma was not necessarily a direct response to stigmatizing behavior and discriminatory attitudes by persons not living with HIV. Furthermore, the absence of a relationship between HIV prevalence and experienced stigma underlines the fact that internalized stigma expressed by PLWH might not be a direct response to experienced stigma. These results align with the discussion presented by Pantelic et al. [32] that internalized HIV stigma may occur without the person having individually experienced discrimination, but rather PLWH can develop HIV-related perceptions, and feel HIV-related stigma, prior to their own diagnosis. Future mixed method research should further explore the relationship between HIV prevalence and stigma expressed by PLWH to understand the underlining mechanisms.

This study has some limitations. First, the PHIA questionnaire used is not the standardized measure used to capture internalized stigma as defined as a situation in which a person living with HIV endorses negative attitudes associated with HIV and accepts them as applicable to themselves [32]. Even though the items used to measure internalized stigma in this work reflect the definition of internalized stigma provided by Earnshaw and Chaudoir [14] and Stangl et al. [24], they were designed to be addressed to the general population rather than specifically to PLWH. Our analysis would benefit by being replicated using a tested measure of internalized stigma design specifically for PLWH to assess if the results found here are persistent. Secondly, although we used multiple items to measure stigma, we were not able to capture all dimensions of HIV-stigma mechanisms. For example, we were not able measure perceived stereotypes, defined as misconceptions that only individuals from some groups can acquire HIV, which is also an important mechanism of stigma among persons not living with HIV with important health-related consequences [14, 24]. Third, although the current study included various types of stigma experiences and stigmatizing practices, many of those constructs were measured using only one item, which could significantly bias results. Future studies should leverage multiple indicators to measure each manifestation of stigma. Finally, due to data availability, the number of countries included in this analysis was limited. Future research should be replicated on larger number of countries with varied HIV contexts.

## Conclusions

This study demonstrated that subnational HIV prevalence had a negative association with stigmatizing behavior, discriminatory attitudes, and prejudice among people not living with HIV towards PLWH, but that HIV prevalence was positively associated with internalized stigma among PLWH. Notably, there was no association between experienced and anticipated stigma expressed by PLWH and HIV prevalence. This indicates that with an increase in HIV prevalence, different aspects of stigma are more impacted on a sub-national level than others, regardless of population sociodemographic factors.

The results of this study have some important policy implications. First, it shows that policies put in place in high prevalence countries to reduce stigma [33] have demonstrated success and should continue and be extended to low prevalence contexts. In fact, though the number of PLWH in this latter context may be low, adverse consequences of stigma [1–5] for people living in those communities may be more pronounced. Additionally, policies put in place to reduce stigma in high prevalence contexts should also target PLWH and address explicitly the issues of internalized stigma, as this can have very deleterious consequences both in terms of mental and physical health, social support and socioeconomic status [8, 10, 14, 24].

This study contributes to the existing literature in many ways. First, to the best of our knowledge it is the first study to assess the link between HIV prevalence and HIV-related stigma distinguishing between the perspective of PLWH and persons not living with HIV from nationally representative data. Second, the study used multiple items to capture the stigma mechanisms suggested by the conceptual work of Earnshaw and Chaudoir [14] and Stangl et al. [24]. Third, contrary to other cross-country analyses, this paper measures HIV prevalence at a subnational level, which allows to account for heterogeneity inside countries, highlighting that people are also influenced by more localized HIV prevalence. Using recent data to contribute to the literature on the link between HIV prevalence and stigma, this paper provides direction on where and how resources should be invested to reduce HIV related stigma and mitigate its potential effects for future populations.

### Supplementary Information

**Additional file 1: Table A1.** Multilevel regression of HIV related stigma among people living without HIV. **Table A2.** Multilevel regression of HIV related stigma among people living with HIV. **Table A3.** Logistic regression of HIV related stigma among people living without HIV with continuous variable of HIV prevalence. **Table A4.** Logistic regression of HIV related stigma among people living with HIV with continuous variable of HIV prevalence.

## Data Availability

Public access PHIA data is available at https://phia.icap.columbia.edu/
