# Supplementary material for: How does HIV-related stigma correlate with HIV prevalence in African countries? Distinct perspectives from individuals living with and living without HIV
Source: BMC Public Health. 2023 Sep 4;23:1720. doi: 10.1186/s12889-023-16545-3 (PMC10478181; doi:10.1186/s12889-023-16545-3)
Supplement: Supplementary file 1 — Additional file 1: Table A1. Multilevel regression of HIV related stigma among people living without HIV. Table A2. Multilevel regression of HIV related stigma among people living with HIV. Table A3. Logistic regression of HIV related stigma among people living without HIV with continuous variable of HIV prevalence. Table A4. Logistic regression of HIV related stigma among people living with HIV with continuous variable of HIV prevalence. [file 12889_2023_16545_MOESM1_ESM.docx]

**Annex**

Table A1: Multilevel regression of HIV related stigma among people living without HIV

|  | (1) |  | (2) |  | (3) |  |
| --- | --- | --- | --- | --- | --- | --- |
|  | Stigmatizing behavior |  | Discriminatory attitude |  | Prejudice |  |
|  | OR | (90% CI) | OR | (90% CI) | OR | (90% CI) |
| *Level 1* |  |  |  |  |  |  |
| Age group: (15-24 years) |  |  |  |  |  |  |
| 25-34 years | 0.67*** | (0.63-0.71) | 0.73*** | (0.67-0.81) | 0.79*** | (0.74-0.84) |
| 35-44 years | 0.59*** | (0.55-0.64) | 0.65*** | (0.59-0.71) | 0.66*** | (0.62-0.70) |
| 45 years + | 0.63*** | (0.56-0.71) | 0.52*** | (0.44-0.61) | 0.68*** | (0.60-0.77) |
| Sex:(Female) |  |  |  |  |  |  |
| Male | 0.88*** | (0.81-0.95) | 1.06 | (1.00-1.13) | 1.06 | (0.99-1.13) |
| Education: (Less than primary) |  |  |  |  |  |  |
| Primary | 0.52*** | (0.49-0.56) | 0.57*** | (0.52-0.62) | 0.86** | (0.77-0.95) |
| Secondary level or higher | 0.23*** | (0.21-0.27) | 0.31*** | (0.28-0.36) | 0.71*** | (0.63-0.80) |
| Wealth quintile: (Lowest) |  |  |  |  |  |  |
| Second | 0.90 | (0.80-1.02) | 0.82*** | (0.73-0.92) | 0.97 | (0.88-1.07) |
| Middle | 0.76*** | (0.69-0.85) | 0.70*** | (0.62-0.79) | 0.85*** | (0.78-0.93) |
| Fourth | 0.58*** | (0.51-0.66) | 0.61*** | (0.54-0.70) | 0.80*** | (0.71-0.89) |
| Highest | 0.45*** | (0.40-0.51) | 0.53*** | (0.44-0.63) | 0.77*** | (0.68-0.87) |
| Urban area:(No) |  |  |  |  |  |  |
| Yes | 0.87* | (0.77-0.99) | 0.82*** | (0.73-0.93) | 0.83*** | (0.75-0.93) |
| *Level 2* |  |  |  |  |  |  |
| Subnational HIV prevalence: (Low) |  |  |  |  |  |  |
| Mid | 0.80** | (0.68-0.96) | 0.87* | (0.78-0.98) | 0.84* | (0.71-0.99) |
| High | 0.65*** | (0.53-0.80) | 0.64*** | (0.56-0.73) | 0.60*** | (0.45-0.80) |
| Proportion with secondary educ. or higher | 1.01 | (1.00-1.01) | 1.02*** | (1.01-1.03) | 1.03*** | (1.03-1.04) |
| Proportion in highest wealth quintile | 1.00 | (0.99-1.00) | 0.99*** | (0.99-1.00) | 0.98*** | (0.97-0.99) |
| Country fixed-effects | Yes |  | Yes |  | Yes |  |
| *Random component* |  |  |  |  |  |  |
| Level 2 variance, 2 (SE) | 0.06 | (0.01) | 0.02 | (0.01) | 0.14 | (0.03) |
| Level 2 intra-class correlation | 0.02 |  | 0.01 |  | 0.4 |  |

Note: Reference category is in parentheses. Significance levels: * p < 0.1, ** p < 0.05, *** p < 0.01. p are nominal p-values and have not been corrected for multiple-hypothesis testing. OR.: Odds ratios, CI: confidence interval. All the estimations are weighted using PHIA individual or knowledge module weights. Reference category in parentheses.

Table A2: Multilevel regression of HIV related stigma among people living with HIV

|  | (1) | |  | (2) | |  | | (3) | |  | |  |
| --- | --- | --- | --- | --- | --- | --- | --- | --- | --- | --- | --- | --- |
|  | Internalized stigma | |  | Experienced discrimination | |  | | Anticipated stigma | |  | |  |
|  | OR | | (90% CI) | OR | | (90% CI) | | OR | | (90% CI) | |  |
| *Level 1* |  | |  |  | |  | |  | |  | |  |
| Age group: (15-24 years) |  | |  |  | |  | |  | |  | |  |
| 25-34 years | 0.62** | | (0.43-0.89) | 0.99 | | (0.34-2.89) | | 0.85 | | (0.55-1.30) | |  |
| 35-44 years | 0.65* | | (0.44-0.96) | 0.78 | | (0.31-1.97) | | 0.66* | | (0.44-0.99) | |  |
| 45 years + | 0.68* | | (0.47-0.99) | 1.07 | | (0.40-2.85) | | 0.58** | | (0.39-0.86) | |  |
| Sex:(Female) |  | |  |  | |  | |  | |  | |  |
| Male | 1.00 | | (0.79-1.27) | 1.36 | | (0.81-2.26) | | 0.99 | | (0.74-1.32) | |  |
| Education: (Less than primary) |  | |  |  | |  | |  | |  | |  |
| Primary | 0.67* | | (0.48-0.95) | 0.53* | | (0.31-0.91) | | 0.84 | | (0.57-1.22) | |  |
| Secondary level or higher | 0.36*** | | (0.20-0.66) | 0.57 | | (0.26-1.22) | | 0.88 | | (0.63-1.24) | |  |
| Wealth quintile: (Lowest) |  | |  |  | |  | |  | |  | |  |
| Second | 0.74 | | (0.53-1.02) | 1.70 | | (0.75-3.86) | | 0.84 | | (0.55-1.27) | |  |
| Middle | 0.72 | | (0.50-1.03) | 1.61 | | (0.71-3.66) | | 0.82 | | (0.55-1.21) | |  |
| Fourth | 0.78 | | (0.52-1.17) | 1.10 | | (0.41-2.92) | | 1.07 | | (0.70-1.63) | |  |
| Highest | 1.31 | | (0.81-2.12) | 1.65 | | (0.58-4.67) | | 1.77** | | (1.13-2.78) | |  |
| Urban area:(No) |  | |  |  | |  | |  | |  | |  |
| Yes | 0.55*** | | (0.41-0.73) | 1.25 | | (0.70-2.23) | | 0.80 | | (0.58-1.09) | |  |
| *Level 2* |  | |  |  | |  | |  | |  | |  |
| Subnational HIV prevalence: (Low) |  | |  |  | |  | |  | |  | |  |
| Mid | 0.88 | | (0.67-1.15) | 1.42 | | (0.75-2.71) | | 1.30 | | (0.97-1.76) | |  |
| High | 1.48* | | (1.02-2.14) | 0.93 | | (0.42-2.08) | | 1.25 | | (0.84-1.86) | |  |
| Proportion with secondary educ. or higher | 0.99 | | (0.97-1.02) | 1.00 | | (0.96-1.03) | | 0.98 | | (0.97-1.00) | |  |
| Proportion in highest wealth quintile | 1.01 | | (0.99-1.02) | 1.00 | | (0.98-1.03) | | 1.01* | | (1.00-1.02) | |  |
| Country: (Zambia) |  | |  |  | |  | |  | |  | |  |
| Malawi | 0.40** | | (0.21-0.74) | 0.81 | | (0.30-2.16) | | 0.30*** | | (0.19-0.49) | |  |
| Tanzania | 0.71 | | (0.42-1.20) | 1.28 | | (0.61-2.67) | | 0.99 | | (0.69-1.43) | |  |
|  |  | |  |  | |  | |  | |  | |  |
| Country fixed-effects | | Yes |  | | Yes | |  | | Yes | |  | |
| *Random component* | |  |  | |  | |  | |  | |  | |
| Level 2 variance, 2 (SE) | | 0.00 | (0.00) | | 0.16 | | (0.03) | | 0.31 | | (0.03) | |
| Level 2 intra-class correlation | | 0.00 |  | | 0.05 | |  | | 0.01 | |  | |

Note: Reference category is in parentheses. Significance levels: * p < 0.1, ** p < 0.05, *** p < 0.01. p are nominal p-values and have not been corrected for multiple-hypothesis testing. OR.: Odds ratios, CI: confidence interval. All the estimations are weighted using PHIA individual or knowledge module weights. Reference category in parentheses.

Table A3: Logistic regression of HIV related stigma among people living without HIV with continuous variable of HIV prevalence.

|  | (1) |  | (2) |  | (3) |  |
| --- | --- | --- | --- | --- | --- | --- |
|  | Stigmatizing behavior |  | Discriminatory attitude |  | Prejudice |  |
|  | OR | (90% CI) | OR | (90% CI) | OR | (90% CI) |
| Age group: (15-24 years) |  |  |  |  |  |  |
| 25-34 years | 0.67*** | (0.61-0.73) | 0.74*** | (0.68-0.81) | 0.76*** | (0.70-0.83) |
| 35-44 years | 0.61*** | (0.55-0.66) | 0.62*** | (0.55-0.70) | 0.64*** | (0.58-0.71) |
| 45 years + | 0.64*** | (0.56-0.74) | 0.58*** | (0.49-0.69) | 0.67*** | (0.58-0.76) |
| Sex:(Female) |  |  |  |  |  |  |
| Male | 0.90** | (0.83-0.97) | 1.05 | (0.97-1.14) | 1.13*** | (1.06-1.20) |
| Education: (Less than primary) |  |  |  |  |  |  |
| Primary | 0.50*** | (0.45-0.56) | 0.58*** | (0.52-0.64) | 0.82*** | (0.74-0.91) |
| Secondary level or higher | 0.23*** | (0.20-0.26) | 0.32*** | (0.28-0.37) | 0.65*** | (0.57-0.75) |
| Wealth quintile: (Lowest) |  |  |  |  |  |  |
| Second | 0.82*** | (0.73-0.91) | 0.74*** | (0.66-0.84) | 0.96 | (0.86-1.06) |
| Middle | 0.69*** | (0.62-0.78) | 0.65*** | (0.57-0.75) | 0.86* | (0.76-0.98) |
| Fourth | 0.54*** | (0.47-0.62) | 0.60*** | (0.51-0.70) | 0.81** | (0.70-0.94) |
| Highest | 0.42*** | (0.35-0.50) | 0.51*** | (0.41-0.62) | 0.80** | (0.68-0.94) |
| Urban area:(No) |  |  |  |  |  |  |
| Yes | 0.85* | (0.74-0.98) | 0.74*** | (0.64-0.85) | 0.76*** | (0.68-0.85) |
| Regional HIV prevalence | 0.96*** | (0.95-0.98) | 0.96*** | (0.95-0.98) | 0.98*** | (0.96-0.99) |
| Proportion with secondary educ. or higher | 1.00 | (0.99-1.01) | 1.01** | (1.00-1.02) | 1.01*** | (1.00-1.02) |
| Proportion in highest wealth quintile | 1.00 | (1.00-1.01) | 1.00 | (0.99-1.00) | 0.99*** | (0.99-1.00) |
| Country: (Zambia) |  |  |  |  |  |  |
| Malawi | 0.26*** | (0.21-0.33) | 0.56*** | (0.45-0.70) | 0.82** | (0.70-0.97) |
| Tanzania | 0.72*** | (0.59-0.87) | 0.96 | (0.80-1.16) | 0.87 | (0.75-1.02) |

Note: Reference category is in parentheses. Significance levels: * p < 0.1, ** p < 0.05, *** p < 0.01. p are nominal p-values and have not been corrected for multiple-hypothesis testing. OR.: Odds ratios, CI: confidence interval. All the estimations are weighted All the estimations are weighted using PHIA individual or knowledge module weights. Reference category in parentheses.

Table A4: Logistic regression of HIV related stigma among people living with HIV with continuous variable of HIV prevalence

|  | (1) |  | (2) |  | (3) |  |
| --- | --- | --- | --- | --- | --- | --- |
|  | Internalized stigma |  | Experienced discrimination |  | Anticipated stigma |  |
|  | OR | (90% CI) | OR | (90% CI) | OR | (90% CI) |
| Mainland or Zanzibar indicator |  |  |  |  |  |  |
| Age group: (15-24 years) |  |  |  |  |  |  |
| 25-34 years | 0.70 | (0.46-1.08) | 0.56 | (0.20-1.60) | 0.80 | (0.51-1.25) |
| 35-44 years | 0.79 | (0.53-1.16) | 0.46 | (0.18-1.22) | 0.63* | (0.41-0.97) |
| 45 years + | 0.87 | (0.55-1.39) | 0.52 | (0.18-1.53) | 0.54** | (0.33-0.90) |
| Sex:(Female) |  |  |  |  |  |  |
| Male | 0.94 | (0.72-1.24) | 1.29 | (0.81-2.05) | 0.95 | (0.68-1.32) |
| Education: (Less than primary) |  |  |  |  |  |  |
| Primary | 0.66* | (0.45-0.95) | 0.81 | (0.38-1.70) | 0.98 | (0.64-1.52) |
| Secondary level or higher | 0.38*** | (0.24-0.63) | 0.73 | (0.31-1.72) | 0.95 | (0.58-1.57) |
| Wealth quintile: (Lowest) |  |  |  |  |  |  |
| Second | 0.58** | (0.38-0.88) | 0.75 | (0.26-2.13) | 0.79 | (0.46-1.37) |
| Middle | 0.62** | (0.42-0.91) | 0.59 | (0.21-1.64) | 0.89 | (0.55-1.47) |
| Fourth | 0.67 | (0.43-1.06) | 0.38 | (0.14-1.05) | 1.04 | (0.64-1.68) |
| Highest | 0.95 | (0.58-1.55) | 0.85 | (0.31-2.37) | 1.85* | (1.08-3.17) |
| Urban area:(No) |  |  |  |  |  |  |
| Yes | 0.60** | (0.41-0.87) | 1.31 | (0.70-2.46) | 0.74 | (0.50-1.07) |
| Regional HIV prevalence | 1.06** | (1.01-1.11) | 1.02 | (0.95-1.10) | 1.04 | (0.99-1.09) |
| Proportion with secondary educ. or higher | 1.00 | (0.97-1.02) | 1.00 | (0.95-1.06) | 0.98 | (0.95-1.00) |
| Proportion in highest wealth quintile | 1.00 | (0.99-1.02) | 0.99 | (0.97-1.02) | 1.02* | (1.00-1.03) |
| Country: (Zambia) |  |  |  |  |  |  |
| Malawi | 0.43* | (0.22-0.87) | 1.00 | (0.28-3.58) | 0.25*** | (0.12-0.51) |
| Tanzania | 0.87 | (0.52-1.47) | 1.90 | (0.51-7.01) | 0.97 | (0.54-1.75) |

Note: Reference category is in parentheses. Significance levels: * p < 0.1, ** p < 0.05, *** p < 0.01. p are nominal p-values and have not been corrected for multiple-hypothesis testing. OR.: Odds ratios, CI: confidence interval. All the estimations are weighted All the estimations are weighted using PHIA individual or knowledge module weights. Reference category in parentheses.
